# Age-structured gametocyte allocation links immunity to epidemiology in malaria parasites

**DOI:** 10.1186/1475-2875-6-123

**Published:** 2007-09-12

**Authors:** Richard E Paul, Sarah Bonnet, Christian Boudin, Timoleon Tchuinkam, Vincent Robert

**Affiliations:** 1 Laboratoire d'Entomologie Médicale, Institut Pasteur de Dakar, 36 Avenue Pasteur, BP 220 Dakar, Sénégal; 2 Laboratoire de Génétique de la réponse aux infections chez l'homme, Institut Pasteur, 28 rue de Dr. Roux, F-75724, Paris cedex 15, France; 3 Laboratoire IRD de Recherche sur le Paludisme, Organisation de Coordination pour la lutte Contre les Endemies en Afrique Centrale, P.O. Box 288, Yaounde, Cameroon; 4 Ecole Nationale Vétérinaire de Nantes, Service de parasitologie, UMR ENVN/INRA 1034 Interactions Hôte-Parasite-Milieu, Atlanpole-La Chantrerie, B.P. 40706, 44307 Nantes cedex 03, France; 5 UR Paludisme Afro-tropical, Institut de Recherche pour le Développement, B.P.1386 Dakar, Sénégal; 6 Institut de Recherche pour le Développement/Muséum National d'Histoire Naturelle, 61 rue Buffon, case courrier 52, 75231 Paris cedex 05, France

## Abstract

**Background:**

Despite a long history of attempts to model malaria epidemiology, the over-riding conclusion is that a detailed understanding of host-parasite interactions leading to immunity is required. It is still not known what governs the duration of an infection and how within-human parasite dynamics relate to malaria epidemiology.

**Presentation of the hypothesis:**

Immunity to *Plasmodium falciparum *develops slowly and requires repeated exposure to the parasite, which thus generates age-structure in the host-parasite interaction. An age-structured degree of immunity would present the parasite with humans of highly variable quality. Evolutionary theory suggests that natural selection will mould adaptive phenotypes that are more precise (less variant) in "high quality" habitats, where lifetime reproductive success is best. Variability in malaria parasite gametocyte density is predicted to be less variable in those age groups who best infect mosquitoes. Thus, the extent to which variation in gametocyte density is a simple parasite phenotype reflecting the complex within-host parasite dynamics is addressed.

**Testing the hypothesis:**

Gametocyte densities and corresponding infectiousness to mosquitoes from published data sets and studies in both rural and urban Cameroon are analysed. The mean and variation in gametocyte density according to age group are considered and compared with transmission success (proportion of mosquitoes infected). Across a wide range of settings endemic for malaria, the age group that infected most mosquitoes had the least variation in gametocyte density, i.e. there was a significant relationship between the variance rather than the mean gametocyte density and age-specific parasite transmission success. In these settings, the acquisition of immunity over time was evident as a decrease in asexual parasite densities with age. By contrast, in an urban setting, there were no such age-structured relationships either with variation in gametocyte density or asexual parasite density.

**Implications of the hypothesis:**

Gametocyte production is seemingly predicted by evolutionary theory, insofar as a reproductive phenotype (gametocyte density) is most precisely expressed (i.e. is most invariant) in the most infectious human age group. This human age group would thus be expected to be the habitat most suitable for the parasite. Comprehension of the immuno-epidemiology of malaria, a requisite for any vaccine strategies, remains poor. Immunological characterization of the human population stratified by parasite gametocyte allocation would be a step forward in identifying the salient immunological pathways of what makes a human a good habitat.

## Background

Mathematical models of infectious diseases and most especially those based on the calculation of R_0_, the basic reproductive number, have proved to be very powerful and robust in interpreting epidemiological trends, most notably for viral and macro-parasitic diseases [1]. However, despite a long history of attempts to model malaria epidemiology, and most especially that of lethal human malaria *Plasmodium falciparum*, the over-riding conclusion is that a detailed understanding of host-parasite interactions leading to immunity is required. The parasite's high degree of genetic diversity and mechanisms of clonal antigenic variation highlight the complex nature of the human-parasite interaction. However, it is still not known what governs the duration of an infection and how within-human parasite dynamics relate to malaria epidemiology. Indeed, controversy still rages over the rate of acquisition of immunity to malaria [2,3].

The classical Ross-Macdonald model (R0=ma2bcμγexp−μT
 MathType@MTEF@5@5@+=feaafiart1ev1aaatCvAUfKttLearuWrP9MDH5MBPbIqV92AaeXatLxBI9gBaebbnrfifHhDYfgasaacH8akY=wiFfYdH8Gipec8Eeeu0xXdbba9frFj0=OqFfea0dXdd9vqai=hGuQ8kuc9pgc9s8qqaq=dirpe0xb9q8qiLsFr0=vr0=vr0dc8meaabaqaciaacaGaaeqabaqabeGadaaakeaacqWGsbGudaWgaaWcbaGaeGimaadabeaakiabg2da9maalaaabaGaemyBa0Maemyyae2aaWbaaSqabeaacqaIYaGmaaGccqWGIbGycqWGJbWyaeaaiiGacqWF8oqBcqWFZoWzaaacbiGae4xzauMae4hEaGNae4hCaa3aaWbaaSqabeaacqGHsislcqWF8oqBcqWGubavaaaaaa@4212@) captures the essence of malaria epidemiology and consists of mosquito components that determine, to a large extent, the force of infection [mosquito density (*m*), biting rate (*a*) and mortality rate (*μ*) and the duration of sporogonic development in the vector (*T*)] and human components that directly relate to the human-parasite interaction. Two of these parameters are concerned with within-host parasite dynamics: the human recovery rate from infection (*γ*) and the infectiousness of an infected individual to mosquitoes (*c*). Parameter (*b*) is the proportion of infective bites that lead to an infection in man.

Immunity to *P. falciparum *develops slowly and requires repeated exposure to the parasite. This generates age-structure in the host-parasite interaction and most notably in the duration of episodes of infection [4]. In areas endemic and stable for malaria, recovery rate is lowest in the young/intermediate age groups [5,6]. Recent studies [2,7], however, suggest that the rate of absolute recovery from infection may not increase with age, even though infecting parasite density may be reduced. Epidemiological models incorporating age-dependency, whether through increased absolute recovery from infection or increased parasite death rate, improve markedly the fit with reality [1]. Measures of human infectiousness to mosquitoes have also shown strong age-specificity such that infections in particular age groups most successfully infect mosquitoes [8-11].

In this work, an attempt is made to identify key measurable parameters linking infection and immunity to epidemiology.

## Presentation of the hypothesis

Age-dependency in the development of an immune response against the asexual parasite stages would present the parasite with habitats of highly variable quality. Evolutionary theory predicts that, in a spatially heterogeneous environment, natural selection will mould adaptive phenotypes such that they are more precise (less variant) in the better and the more frequent habitats and less so in infrequent habitats and those where reproductive performance is poor [12], i.e. natural selection will act on both the mean and the variance of the phenotype in question. Heterogeneity in habitat quality can severely alter R_0 _and it is trivial to note that optimising transmission (*c*) in the age groups where recovery rate (*γ*) is the lowest will significantly increase R_0_. If the parasite is indeed optimising transmission according to the immune-dependent quality of individuals, this should be apparent from the within-host parasite dynamics and notably that pertaining to gametocyte production.

## Testing the hypothesis

Gametocyte densities and corresponding infectiousness to mosquitoes from published data sets [9,10,13] and studies in both rural [11,14] and urban [15] settings in Cameroon are examined. The mean and variation in gametocyte density (as proxies of the parasite's transmission phenotype) according to age group are considered and compared with actual measures of transmission success (the proportion of mosquitoes that become infected). Maturation of gametocytes occurs slowly and infections with apparent gametocytes may not be infectious at that exact time but may be so hours later. Therefore, all observed gametocyte densities are included. The age groups chosen in the published and the rural Cameroon data sets reflect the classical age distribution as defined by malaria prevalence rates that provide a gross measure of the force of infection and gradual development of immunity. The decrease in asexual parasite densities with age is a more sensitive indication of the slow acquisition of immunity that generates age structure and is thus presented. By contrast, malaria transmission in urban settings is low and unstable, resulting in a poor acquisition of immunity. Therefore, age-stratification of the urban Cameroon data set would not be expected to reflect age-specific development of immunity or be apparent from asexual parasite densities.

### Cameroon study sites and data collection

The study sites and methods have been previously published [11,14,15]. Briefly, in the rural setting, individuals, symptomatic or not, were recruited in two adjacent villages in the district of Mengang, South Cameroon, in an area of degraded forest with seasonal transmission (intensity of 170 infected bites/person per year) and hyperendemic malaria (*P. falciparum *prevalence rates range from 49% to 82%, depending on the season). Mosquitoes were gorged directly on individuals whether positive or not for gametocytes. In the urban setting, patients were recruited at a Public Health Centre in central Yaounde, the capital town of Cameroon. From those individuals positive for gametocytes by thick blood smear, venous blood was immediately placed into a membrane feeder upon which mosquitoes gorged. Previous comparative studies have shown that although direct feeding of mosquitoes on infectious individuals yields higher infections in mosquitoes than feeding through a membrane, there is good concordance between the two tests for both mosquito infection percentages and oocyst loads [16,17]. In both studies, only experiments where at least 20 mosquitoes survived to day of dissection for oocyst counts (day 7 post-gorging) were used in analyses. The mean number of mosquitoes dissected per experiment was 35 and 36 in the rural and urban settings respectively. Blood parasite counts were established by thick smear per 1,000 leucocytes for asexual parasite density and 1,000 and 3,000 leucocytes for gametocytes respectively in urban and rural settings, assuming an average number of 8,000 leucocytes per microlitre of blood. The studies used the same laboratory mosquito strain reared in the same insectary (*Anopheles gambiae *s.s. Yaounde strain; OCEAC insectary).

### Statistical analyses

The effects of age group and gametocyte density per infection on transmission success in the Cameroon data sets were analysed in Genstat version 7 using logistic regression specifying a binomial error structure. The effect of age group on asexual parasite density was analysed by loglinear regression fitting a GLM, specifying a Poisson error structure. Dispersion parameters were estimated and thus the analyses generate an F statistic in the analysis of deviance.

Using data from the Cameroon studies as well as the two published studies, how the mean and the variability in gametocyte density per age group affected transmission success were then examined. This combined data set was analysed by two alternative methods: a REML meta-analysis and by fitting a GLMM (Mixed model) with study specified in the random model and the mean proportion of mosquitoes infected by age group as the response variate. A REML meta-analysis takes into account differences between sites by incorporating the residual variance of each experiment. GLMMs account for variation due to study site and calculate the variance component due to this random factor independently of that due to the factor of interest – i.e. the response variate. Because the measure of variability in gametocyte density per age group was previously given as confidence intervals (CI) in the published studies, this measure was used for the Cameroon data sets. The lower CI subtracted from the upper CI was calculated to generate a value of dispersion in gametocyte density by age group. Both this value and mean gametocyte density per age group were normalized for each study by dividing by the lowest value, thus giving the lower value of 1 per study. Such normalization enables comparison of the general effect of gametocyte density and variability in density by age group without study specific effects, most evident of which are the very different overall gametocyte densities. Both the normalized gametocyte density mean and variability were then fitted as explanatory variates. Because the data were over-dispersed, a dispersion parameter was estimated. Statistical significance is presented as Wald statistics, which approximate to a *χ*^2 ^distribution. For the Cameroon data sets, variance/mean ratios and the Standardized Morisita index (I_p_) were additionally calculated. Sample size influences both confidence intervals and variances. The larger the sample size the smaller these measures will be for a data set of the same intrinsic variability. The Standardized Morisita index (I_p_), however, is considered the best measure of dispersion being independent of sample size [18]. To calculate this index, the Morisita's index of dispersion (I_d_) and two critical values, the Uniform index (M_u_) and the clumped index (M_c_) are calculated as follows:

Id=n[∑x2−∑x(∑x)2−∑x]
 MathType@MTEF@5@5@+=feaafiart1ev1aaatCvAUfKttLearuWrP9MDH5MBPbIqV92AaeXatLxBI9gBaebbnrfifHhDYfgasaacH8akY=wiFfYdH8Gipec8Eeeu0xXdbba9frFj0=OqFfea0dXdd9vqai=hGuQ8kuc9pgc9s8qqaq=dirpe0xb9q8qiLsFr0=vr0=vr0dc8meaabaqaciaacaGaaeqabaqabeGadaaakeaacqWGjbqsdaWgaaWcbaGaemizaqgabeaakiabg2da9iabd6gaUnaadmaabaWaaSaaaeaadaaeabqaaiabdIha4naaCaaaleqabaGaeGOmaidaaOGaeyOeI0YaaabqaeaacqWG4baEaSqabeqaniabggHiLdaaleqabeqdcqGHris5aaGcbaWaaeWaaeaadaaeabqaaiabdIha4bWcbeqab0GaeyyeIuoaaOGaayjkaiaawMcaamaaCaaaleqabaGaeGOmaidaaOGaeyOeI0YaaabqaeaacqWG4baEaSqabeqaniabggHiLdaaaaGccaGLBbGaayzxaaaaaa@47C2@

Mu=χ.9752−n+∑x(∑x)−1
 MathType@MTEF@5@5@+=feaafiart1ev1aaatCvAUfKttLearuWrP9MDH5MBPbIqV92AaeXatLxBI9gBaebbnrfifHhDYfgasaacH8akY=wiFfYdH8Gipec8Eeeu0xXdbba9frFj0=OqFfea0dXdd9vqai=hGuQ8kuc9pgc9s8qqaq=dirpe0xb9q8qiLsFr0=vr0=vr0dc8meaabaqaciaacaGaaeqabaqabeGadaaakeaacqWGnbqtdaWgaaWcbaGaemyDauhabeaakiabg2da9maalaaabaacciGae83Xdm2aa0baaSqaaiabc6caUiabiMda5iabiEda3iabiwda1aqaaiabikdaYaaakiabgkHiTiabd6gaUjabgUcaRmaaqaeabaGaemiEaGhaleqabeqdcqGHris5aaGcbaWaaeWaaeaadaaeabqaaiabdIha4bWcbeqab0GaeyyeIuoaaOGaayjkaiaawMcaaiabgkHiTiabigdaXaaaaaa@4515@

Mc=χ.0252−n+∑x(∑x)−1
 MathType@MTEF@5@5@+=feaafiart1ev1aaatCvAUfKttLearuWrP9MDH5MBPbIqV92AaeXatLxBI9gBaebbnrfifHhDYfgasaacH8akY=wiFfYdH8Gipec8Eeeu0xXdbba9frFj0=OqFfea0dXdd9vqai=hGuQ8kuc9pgc9s8qqaq=dirpe0xb9q8qiLsFr0=vr0=vr0dc8meaabaqaciaacaGaaeqabaqabeGadaaakeaacqWGnbqtdaWgaaWcbaGaem4yamgabeaakiabg2da9maalaaabaacciGae83Xdm2aa0baaSqaaiabc6caUiabicdaWiabikdaYiabiwda1aqaaiabikdaYaaakiabgkHiTiabd6gaUjabgUcaRmaaqaeabaGaemiEaGhaleqabeqdcqGHris5aaGcbaWaaeWaaeaadaaeabqaaiabdIha4bWcbeqab0GaeyyeIuoaaOGaayjkaiaawMcaaiabgkHiTiabigdaXaaaaaa@44D5@

where n is the sample size, *x *is the number of gametocytes in each individual, χ.9752
 MathType@MTEF@5@5@+=feaafiart1ev1aaatCvAUfKttLearuWrP9MDH5MBPbIqV92AaeXatLxBI9gBaebbnrfifHhDYfgasaacH8akY=wiFfYdH8Gipec8Eeeu0xXdbba9frFj0=OqFfea0dXdd9vqai=hGuQ8kuc9pgc9s8qqaq=dirpe0xb9q8qiLsFr0=vr0=vr0dc8meaabaqaciaacaGaaeqabaqabeGadaaakeaaiiGacqWFhpWydaqhaaWcbaGaeiOla4IaeGyoaKJaeG4naCJaeGynaudabaGaeGOmaidaaaaa@3361@ and χ.0252
 MathType@MTEF@5@5@+=feaafiart1ev1aaatCvAUfKttLearuWrP9MDH5MBPbIqV92AaeXatLxBI9gBaebbnrfifHhDYfgasaacH8akY=wiFfYdH8Gipec8Eeeu0xXdbba9frFj0=OqFfea0dXdd9vqai=hGuQ8kuc9pgc9s8qqaq=dirpe0xb9q8qiLsFr0=vr0=vr0dc8meaabaqaciaacaGaaeqabaqabeGadaaakeaaiiGacqWFhpWydaqhaaWcbaGaeiOla4IaeGimaaJaeGOmaiJaeGynaudabaGaeGOmaidaaaaa@3345@ are the values of the chi-squared with (*n*-1) degrees of freedom that have 97.5% or 2.5% of the area to the right. The Standardized Morisita index (I_p_) is then calculated by one of the four following formulae:

(a) when I_d _≥ M_c _> 1, Ip=0.5+0.5×(Id−Mcn−Mc)
 MathType@MTEF@5@5@+=feaafiart1ev1aaatCvAUfKttLearuWrP9MDH5MBPbIqV92AaeXatLxBI9gBaebbnrfifHhDYfgasaacH8akY=wiFfYdH8Gipec8Eeeu0xXdbba9frFj0=OqFfea0dXdd9vqai=hGuQ8kuc9pgc9s8qqaq=dirpe0xb9q8qiLsFr0=vr0=vr0dc8meaabaqaciaacaGaaeqabaqabeGadaaakeaacqWGjbqsdaWgaaWcbaGaemiCaahabeaakiabg2da9iabicdaWiabc6caUiabiwda1iabgUcaRiabicdaWiabc6caUiabiwda1iabgEna0oaabmaabaWaaSaaaeaacqWGjbqsdaWgaaWcbaGaemizaqgabeaakiabgkHiTiabd2eannaaBaaaleaacqWGJbWyaeqaaaGcbaGaemOBa4MaeyOeI0Iaemyta00aaSbaaSqaaiabdogaJbqabaaaaaGccaGLOaGaayzkaaaaaa@45C3@, (b) when M_c _> I_d _≥ 1, Ip=0.5×(Id−1Mu−1)
 MathType@MTEF@5@5@+=feaafiart1ev1aaatCvAUfKttLearuWrP9MDH5MBPbIqV92AaeXatLxBI9gBaebbnrfifHhDYfgasaacH8akY=wiFfYdH8Gipec8Eeeu0xXdbba9frFj0=OqFfea0dXdd9vqai=hGuQ8kuc9pgc9s8qqaq=dirpe0xb9q8qiLsFr0=vr0=vr0dc8meaabaqaciaacaGaaeqabaqabeGadaaakeaacqWGjbqsdaWgaaWcbaGaemiCaahabeaakiabg2da9iabicdaWiabc6caUiabiwda1iabgEna0oaabmaabaWaaSaaaeaacqWGjbqsdaWgaaWcbaGaemizaqgabeaakiabgkHiTiabigdaXaqaaiabd2eannaaBaaaleaacqWG1bqDaeqaaOGaeyOeI0IaeGymaedaaaGaayjkaiaawMcaaaaa@400E@,

(c) when 1 > I_d _> M_u_, Ip=−0.5×(Id−1Mu−1)
 MathType@MTEF@5@5@+=feaafiart1ev1aaatCvAUfKttLearuWrP9MDH5MBPbIqV92AaeXatLxBI9gBaebbnrfifHhDYfgasaacH8akY=wiFfYdH8Gipec8Eeeu0xXdbba9frFj0=OqFfea0dXdd9vqai=hGuQ8kuc9pgc9s8qqaq=dirpe0xb9q8qiLsFr0=vr0=vr0dc8meaabaqaciaacaGaaeqabaqabeGadaaakeaacqWGjbqsdaWgaaWcbaGaemiCaahabeaakiabg2da9iabgkHiTiabicdaWiabc6caUiabiwda1iabgEna0oaabmaabaWaaSaaaeaacqWGjbqsdaWgaaWcbaGaemizaqgabeaakiabgkHiTiabigdaXaqaaiabd2eannaaBaaaleaacqWG1bqDaeqaaOGaeyOeI0IaeGymaedaaaGaayjkaiaawMcaaaaa@40FB@ and (d) when 1 > M_u _> I_d_, Ip=−0.5+0.5×(Id−MuMu)
 MathType@MTEF@5@5@+=feaafiart1ev1aaatCvAUfKttLearuWrP9MDH5MBPbIqV92AaeXatLxBI9gBaebbnrfifHhDYfgasaacH8akY=wiFfYdH8Gipec8Eeeu0xXdbba9frFj0=OqFfea0dXdd9vqai=hGuQ8kuc9pgc9s8qqaq=dirpe0xb9q8qiLsFr0=vr0=vr0dc8meaabaqaciaacaGaaeqabaqabeGadaaakeaacqWGjbqsdaWgaaWcbaGaemiCaahabeaakiabg2da9iabgkHiTiabicdaWiabc6caUiabiwda1iabgUcaRiabicdaWiabc6caUiabiwda1iabgEna0oaabmaabaWaaSaaaeaacqWGjbqsdaWgaaWcbaGaemizaqgabeaakiabgkHiTiabd2eannaaBaaaleaacqWG1bqDaeqaaaGcbaGaemyta00aaSbaaSqaaiabdwha1bqabaaaaaGccaGLOaGaayzkaaaaaa@44A6@.

The Standardized Morisita index (I_p_) ranges from -1 to +1. Random patterns give a value of zero, clumped patterns above zero and uniform patterns below zero. With respect to gametocyte data, the higher the value above zero the more over-dispersed, and thus more variable, are the gametocyte densities.

## Results

**Figure 1:**
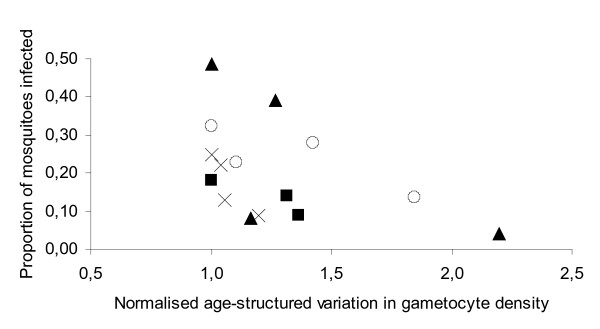
**Age group variation in gametocyte density with corresponding infectiousness to mosquitoes**. Data shown in Table 1. The higher minus the lower 95% confidence intervals are taken as a measure of gametocyte density variation and are normalized within each study area by dividing by the lowest value, thus giving the lower value of 1. Circles – *P. vivax *Sri Lanka [9]; Triangles – *P. falciparum *Sri Lanka [9]; Squares – *P. falciparum *Kenya [10]; Crosses – *P. falciparum *Cameroon [11].

Mosquito infection rates have been classically shown to correlate positively with individual gametocyte density. This positive correlation was confirmed here for the Cameroon rural data set, including only those individuals who infected at least one mosquito (F_1,45 _= 13.9 P < 0.001 N = 50). However, gametocyte density *per se *explained only 16.6% of the variation in transmission success and there was a significant effect of age group on mosquito infection rates (F_3,45 _= 3.43, P = 0.025 N = 50). Within any of the published data sets in Kenya and Sri Lanka and the rural Cameroon data set, the mean gametocyte densities were relatively constant across age groups (Table 1) and typical of those observed in rural endemic situations across the globe. It is notable that the most infectious age groups in any of these settings were not those with the highest mean gametocyte densities (Table 1). Indeed, mean gametocyte density per age group did not correlate with mosquito infection rates (GLMM *χ*^2^_1 _= 1.03, P = 0.31). By contrast, it is striking that the age groups that had the lowest variability in gametocyte density infected the highest proportion of mosquitoes (Figure 1) (REML meta-analysis: *χ*^2^_1 _= 7.95, P = 0.005; GLMM *χ*^2^_1 _= 6.72, P = 0.01). Variation in gametocyte density (i.e. gametocyte precision) explained 17.1% of variation in age group transmission success. Using the rural Cameroon data set, this relationship was confirmed when measuring gametocyte variability using several different dispersion indices (Table 1), including the Standardized Morisita index (I_p_), which is considered independent of sample size [18]; all dispersion indices show the same pattern in the Cameroon data set. Moreover, Table 1 shows that, with the exception of *P. vivax *gametocyte data from Sri Lanka, gametocyte variability is lower in the best transmitting age group rather than the age group with the largest sample size. Larger sample sizes would be expected to yield lower values of dispersion if intrinsic variability were the same among groups and could therefore explain degrees of dispersion. Here this is not the case.

**Table 1:** Age-specific gametocyte densities with measures of transmission success in rural Cameroon [11] and published data sets [9,10].

		Gametocyte density						
				
Study Site	age group	N	Geometric mean/*μ*l (± CI_95%_)	Normalized _95%_Confidence Intervals	Standardized Morisita Index	Variance/mean	Mean proportion mosquitoes infected ± s.e. (n)	Proportional contribution to human infectious reservoir
Cameroon	0–5	40	3.56 (0.8–6.32)	1.04	0.521	12.4	0.22 ± 0.05 (17)	0.32
	6–9	36	3.54 (0.88–6.2)	**1.00**	**0.518**	**10.0**	**0.25 ± 0.05** **(14)**	**0.43**
	10–15	28	2.84 (0.04–5.64)	1.05	0.531	11.6	0.13 ± 0.06 (7)	0.11
	15+	33	*3.71* *(0.64–6.88)*	1.20	0.528	13.6	0.09 ± 0.04 (12)	0.10

Kenya	1–4	33	29.3 (27.1–31.7)	**1.00**			**0.16 (20)**	**0.38**
	5–9	36	*30.7* *(27.8–33.8)*	1.33			0.14 (20)	0.34
	10–14	17	29 (26.0–32.9)	1.50			0.07 (18)	0.09

			Gametocytes/red blood cells (×10^-2^) ± SD					

Sri Lanka	0–5	47	*2.2 ± 3*	1.85			0.14	0.11
*P. vivax*	6–15	122	1.3 ± 2	**1.00**			**0.32**	0.27
	16–25	75	1.9 ± 2	1.42			0.28	**0.32**
	26–50	82	1.4 ± 2	1.11			0.23	0.28

*P. falciparum*	0–5	26	0.07 ± 0.3	1.17			0.08	0.01
	6–20	130	0.09 ± 0.4	**1.00**			**0.49**	**0.68**
	21–50	187	0.13 ± 0.5	1.27			0.39	0.30
	50+	27	*0.16 ± 0.5*	2.20			0.04	0.01

Differences in the upper and lower 95% confidence intervals are normalized within each study by dividing by the lowest value. The proportional contribution to the infectious reservoir is the proportion of an age group that are infectious to mosquitoes multiplied by the proportion of mosquitoes that become infected. In bold, values for the least dispersed gametocyte densities and the highest mosquito infection rates; in *italics *the highest gametocyte densities. N is the number of individuals with gametocyte parasites and n is the number of individuals infecting mosquitoes.

Immune-generated age-structure is apparent in rural Cameroon, where asexual parasite densities (and variation in density) decreased significantly with age from a mean of 3965 parasites/*μ*l (SE ± 690) in the youngest age group to 829 parasites/*μ*l (SE ± 168) in the oldest age group (F_3,410 _= 11.51 P < 0.001 N = 413)(Table 2). Likewise, the prevalence of infection and the proportion of infections bearing gametocytes also decreased significantly in the eldest age group [11]. By contrast in the urban setting there was no effect of age group on asexual parasite densities (F_4,90 _= 0.68 P = 0.68, N = 95), with a high overall mean of 7626 parasites/*μ*l (SE ± 1013) (Table 2), as would be expected if acquisition of immunity were low. This lack of age-structure is mirrored in mosquito infection rates where there was no effect of age (F_4,80 _= 1.3, P = 0.27, N = 86), nor was there any relationship between gametocyte variability and transmission in the urban sample (Table 3) (F_1,3 _= 0.12 P = 0.752). However, there was a strong effect of gametocyte density *per se *(F_1,84 _= 23.1 P < 0.001 N = 86), which accounted for 28.1% of variation in mosquito infection rates.

**Table 2:** Age-specific asexual *Plasmodium *spp. prevalence rates and densities.

Study Site	age group	Trophozoite prevalence (%)	N	Mean trophozoite density (± CI_95%_)
Cameroon	0–5	66.4	42	3965 (2613–5317)
Rural	6–9	71	119	1726 (1199–2253)
	10–15	69.5	104	1387 (879–1895)
	15+	39.5	148	829 (500–1158)

Cameroon	5–10	NA	14	7600 (2720–12480)
Urban	11–14		25	8471 (4616–12326)
	15–20		20	9721 (5103–14339)
	21–25		19	5842 (2169–9515)
	25+		17	5934 (2020–9848)

Kenya	1–4	89	63	1603 (968–2654)
	5–9	94	83	654 (444–963)
	10–14	90	88	188 (134–264)

		Trophozoite Incidence (%)		

Sri Lanka				
*P. vivax*	0–5	10	753	NA
	6–15	13.8	829	
	16–25	9.4	712	
	26–50	6.5	1080	
	50+	3.9	251	
*P. falciparum*	0–5	7.4	753	
	6–15	20.1	829	
	16–25	17.4	712	
	26–50	12.8	1080	
	50+	7.6	251	

N is the number of individuals contributing to the calculation of mean trophozoite densities or, for the case of Sri Lanka, the incidence rates. NA is Not Available.

**Table 3:** Age-specific gametocyte densities with measures of transmission success in urban Cameroon [15].

	Gametocyte density						
			
age group	N	Geometric mean/*μ*l (± CI_95%_)	Normalized _95%_Confidence Intervals	Standardized Morisita Index	Variance/mean	Mean proportion of mosquitoes infected ± s.e. (n)	Proportional contribution to human infectious reservoir
5–10	26	78.2 (± 51.2)	**1.00**	**0.518**	**129.7**	0.2 ± 0.05 (17)	0.23
11–14	24	*118.7 (± 119.8)*	2.34	0.536	369.6	0.16 ± 0.04 (15)	0.17
15–20	26	107.2 (± 93.4)	1.82	0.528	290.0	**0.24 ± 0.04 (14)**	0.22
21–25	33	76.5 (± 52.4)	1.02	0.519	168.1	0.21 ± 0.05 (24)	**0.26**
25+	29	48.9 (± 59.4)	1.16	0.539	233.6	0.12 ± 0.03 (16)	0.11

Differences in the upper and lower 95% confidence intervals are normalized by dividing by the lowest value. The proportional contribution to the infectious reservoir is the proportion of an age group that are infectious to mosquitoes multiplied by the proportion of mosquitoes that become infected. In bold, values for the least dispersed gametocyte densities and the highest mosquito infection rates; in *italics *the highest gametocyte densities. N is the number of individuals with gametocyte parasites and n is the number of individuals infecting mosquitoes. No age was recorded for 4 of the 90 urban individuals infecting mosquitoes.

## Implications of the hypothesis

These analyses reveal a significant relationship between precision in gametocyte density and the transmission success of age groups in endemic settings of malaria transmission. Importantly, the actual age group most infectious to mosquitoes varied according to the transmission intensity, becoming younger with increasing intensity. Mean gametocyte densities by age group were not, however, related to transmission success except in urban Cameroon. As expected in endemic conditions, an age-dependent degree of acquired immunity was evident from the gradual decrease in mean asexual parasite densities. By contrast, the relationship between gametocyte variability and transmission success did not occur in an urban setting where individuals of all ages did not differ in their overall degree of immunity, as apparent from their similar asexual parasite densities.

Studies to date have examined human infectiousness to mosquitoes at three distinct levels: (1) Infectiousness of the individual according to gametocyte density [e.g. [19]], (2) the age group reservoir of infection within a site [e.g. [8,11,20]], and most recently (3) across populations of differing intensity [21]. Here, a gametocyte phenotype (low variance of gametocyte density) measured at the level of age group, but which incorporates individual infection data, is consistently related to transmission success across populations from differing transmission intensities. This relationship is consistent with the thesis that natural selection should mould phenotypes (gametocyte density) such that they are less variant in the most productive habitat. Why certain age groups are the most productive (i.e. infectious) is less clear. Gametocyte epidemiology is complex but does display some age-structure (For an excellent review see [22]). In semi-immune populations, gametocyte prevalence does not always decrease with age [20] and, when it does, tends to reflect age-asexual parasite prevalence profiles [8,10,23]. This suggests that low gametocyte prevalence reflects the development of immunity against the asexual stages [24] rather than any effective anti-gametocyte immune response. However, a recent reappraisal suggests that gametocyte prevalence may decrease more rapidly with age than asexual parasite prevalence rates and that there might be additional anti-gametocyte immune responses [22]. Moreover, when gametocytes are present in older age groups, their densities, relative to the asexual parasite density whence they arise, are generally increased [22]. This is consistent with the known influence of both specific and non-specific anti-asexual parasite immune mechanisms on the rate of conversion from asexual to gametocyte stage parasites [25-27].

How such variation in gametocyte prevalence rates and densities per human age group relate to transmission success is unclear. Although increasing gametocyte density tends to result in greater infectiousness to mosquitoes [19], it has been repeatedly demonstrated that high gametocyte densities do not guarantee high mosquito infection rates [15,19,20]. Recent work within the context of developing a transmission-blocking vaccine has highlighted concurrent gametocyte density, but not age, as being positively correlated with transmission-blocking immunity [28]. Not unsurprisingly, there exists a minimum gametocyte density necessary for effective transmission, but one which is variable according to study site [29]. Low asexual parasite densities, such as those found in older semi-immune individuals, might therefore generate insufficient gametocytes for transmission. In the rural Cameroon data set here, however, mean and even mode gametocyte densities did not differ among age groups. Conversely, high asexual parasite densities, such as those seen in infants, generate an excess of gametocytes [30] and thus may induce transmission blocking anti-gametocyte specific immunity. Moreover, concurrent fever, induced by high asexual parasitaemia, reduces infectivity [31,32]. An age-dependent pyrogenic threshold of *P. falciparum *parasitaemia has been demonstrated [33]; notably the individuals most tolerant of parasite density were neither the very young nor the older age groups. Such a threshold is expected to vary according to the transmission intensity. This might be an explanation for our observed differences in age of the best-transmitting groups in areas of differing transmission intensity.

Although age-specific transmission success could be explained with reference to age-specific pyrogenic thresholds, it is not clear why variation in gametocyte density should correlate similarly with age. Variation in asexual parasitaemia was found to decrease with age as might be expected with the development of immunity and yet variation in gametocyte density did not. Although variation in gametocyte density in the youngest age groups may thus be explained as a consequence of the variation in asexual parasite densities, the same argument can not simultaneously explain the increased variability in the older age groups. Specific anti-asexual parasite immunity may additionally be involved in older age groups acting indirectly in conflicting ways on gametocyte density: both reducing the asexual parasite source and increasing the rate of conversion to gametocytes. This dual effect on gametocyte density could generate increased variability in both gametocyte density and transmission success. Potentially there exists a hitherto unidentified anti-gametocyte specific response that increases with the acquisition of anti-parasite (asexual) immunity.

Hitherto unmentioned, is the documented relationship between gametocyte production and haematological factors [34-36]. Although oxidative stress by cytokine response to infection can lead to alteration of uninfected RBC membranes and thus enhance their removal by the spleen [37], to date no age-specific anti-parasite immune response affecting haemolysis and hence erythropoiesis has been described [38]. Indeed, in regions of high intensity, anaemia occurs in infants and in certain risk groups (pregnant women); older age groups do not apparently suffer from malaria-induced anaemia though haematopoietic activity has not been surveyed as such across a population.

Malaria parasite gametocyte allocation has proven highly informative, revealing adaptive strategies that optimize transmission according to parasite population structure [39,40] and to ensure transmission success [41-43]. Here, examination of variation in gametocyte density reveals a measure that captures the essence of the parasite's behaviour with respect to transmission. The parasite has a very precise gametocyte allocation in individuals of an age from whom transmission to mosquitoes is best. This suggests that the parasite is best adapted to specific age groups, i.e. the parasite is being more precise in the better habitat.

The human immune response characterizes habitat quality of humans for malaria parasites. Infection longevity would be expected to be beneficial for the parasite and thus those individuals with the longest duration of infection be predicted to be the highest quality habitats. The duration of an infection has proved, however, a very difficult parameter to measure and recently the subject of renewed discussion [2,3,7]. Nevertheless, estimates made in areas of high transmission intensity suggest that the longest duration occurs in intermediate age groups [5,6]; it is not known, however, how this relates to transmission success or what are the principle immunological mechanisms involved. Immuno-tolerance is an accepted feature of malaria parasite sustained infection and a role for the innate immune response has been recently proposed [44]. The age groups most infectious to mosquitoes are those who seemingly have the lowest recovery rate from infection [5,6], when extrapolating from studies in regions of similar transmission intensity to the Kenyan and Cameroon study examined here [10,11]. The longer duration of infection in individuals apparently tolerant to increased parasite densities may provide sufficient time, in a relatively stable environment, with which to produce gametocytes. This would provide the link between immune-dependent habitat quality and transmission success.

Malaria parasite epidemiology may be best understood with reference to reproductive allocation. A deeper understanding of the immunological basis to the host-parasite interaction and what defines a "better host" will be more readily achieved by inspection of host anti-parasite immune responses in age groups defined by the reproductive precision of their infecting parasites. Importantly, the best human habitats were those who had had previous exposure to the parasite. Vaccine strategies designed to reduce morbidity in infants may be at risk of generating of better quality individuals for parasite transmission. In the light of the current widespread vaccine trials this must be carefully monitored to avoid undesirable side-effects.

## Authors' contributions

RELP conceived the idea, analyzed the data and wrote the paper. SB & CB performed all the data collection in rural Cameroon, made comments on and edited the paper. TT & VR performed all the data collection in Yaounde, TT made comments on and edited the paper and VR co-wrote sections of the paper.
